# Prevalence of Clinically Relevant Germline *BRCA* Variants in a Large Unselected South African Breast and Ovarian Cancer Cohort: A Public Sector Experience

**DOI:** 10.3389/fgene.2022.834265

**Published:** 2022-04-08

**Authors:** Nerina C. Van der Merwe, Herkulaas MvE Combrink, Kholiwe S. Ntaita, Jaco Oosthuizen

**Affiliations:** ^1^ Division of Human Genetics, National Health Laboratory Service, Bloemfontein, South Africa; ^2^ Division of Human Genetics, Faculty of Health Sciences, University of the Free State, Bloemfontein, South Africa; ^3^ Economic and Management Sciences, University of the Free State, Bloemfontein, South Africa; ^4^ Interdisciplinary Centre for Digital Futures, University of the Free State, Bloemfontein, South Africa

**Keywords:** familial breast and ovarian cancer, predisposing genes *BRCA1/2*, central South Africa, National Health Laboratory Service, public sector

## Abstract

Breast cancer is a multifaceted disease that currently represents a leading cause of death in women worldwide. Over the past two decades (1998–2020), the National Health Laboratory Service’s Human Genetics Laboratory in central South Africa screened more than 2,974 breast and/or ovarian cancer patients for abnormalities characteristic of the widely known familial breast cancer genes, Breast Cancer gene 1 (*BRCA1*) and Breast Cancer gene 2 (*BRCA2*)*.* Patients were stratified according to the presence of family history, age at onset, stage of the disease, ethnicity and mutation status relative to *BRCA1/2*. Collectively, 481 actionable (likely-to pathogenic) variants were detected in this cohort among the different ethnic/racial groups. A combination of old (pre-2014) and new (post-2014) laboratory techniques was used to identify these variants. Additionally, targeted genotyping was performed as translational research revealed the first three recurrent South African pathogenic variants, namely *BRCA1* c.1374del (legacy name 1493delC), *BRCA1* c.2641G>T (legacy name E881X) and *BRCA2* c.7934del (legacy name 8162delG). This initial flagship study resulted in a cost-effective diagnostic test that enabled screening of a particular ethnic group for these variants. Since then, various non-Afrikaner frequent variants were identified that were proven to represent recurrent variants. These include *BRCA2* c.5771_5774del (legacy name 5999del4) and *BRCA2* c.582G>A, both Black African founder mutations. By performing innovative translational research, medical science in South Africa can adopt first-world technologies into its healthcare context as a developing country. Over the past two decades, the progress made in the public sector enabled a pivotal shift away from population-directed genetic testing to the screening of potentially all breast and ovarian cancer patients, irrespective of ethnicity, family history or immunohistochemical status. The modifications over the years complied with international standards and guidelines aimed at universal healthcare for all. This article shares all the cohort stratifications and the likely-to pathogenic variants detected.

## 1 Introduction

The two familial breast cancer (BC) genes, Breast Cancer gene 1 (*BRCA1*) and Breast Cancer gene 2 (*BRCA2*), are highly penetrant and contribute to various cellular events ranging from the response to DNA damage to control of the cell cycle and apoptosis (Yoshida and Miki, 2004). Germline pathogenic variants in these genes create genetic instability impacting their capacity to repair the damage. Likely- to pathogenic variants in these genes are associated with hypersensitivity in BC patients. Their presence results in potentially severe radiotherapy complications during treatment due to spontaneous and enhanced radio-sensitivity (Chistiakov et al., 2008; Kan and Zhang, 2015). Disruptive *BRCA1/2* variants are associated with a predisposition to breast and ovarian cancer, and although at a lower frequency, prostate, pancreas and other cancer types are also linked to pathogenic *BRCA1/2* variants. Although these two high-impact cancer-predisposing genes were discovered more than two decades ago, they have dominated the field of BC genetics ever since.

BC is the most common cancer and the leading cause of cancer-related death in females worldwide (Bray et al., 2018). Most cases are sporadic. However, 5%–10% can be attributed to a hereditary component (Larsen et al., 2014). The disease was mostly considered an illness of the affluent; however, the incidence in developing countries, such as South Africa (SA), is rapidly increasing (Joffe et al., 2018). The age-standardized annual BC incidence rate (ASR) per 100,000 ranges between 52.92 and 79.3 for Asian, Caucasian and mixed-race SA women, compared to 29.1 for Black women. The average ASR, however, is currently 1 in 32 for a SA female to develop the disease (Francies et al., 2015).

The complex history of sub-Saharan Africa has highlighted the diverse populations of SA regarding the field of medical and population genetics (Oosthuizen et al., 2021). Although SA harbors over 60 million people, each of its main population groups has a unique origin. This diversity resulted from various migration events from all over the globe, such as European colonialism from predominantly north-western Europe, which gave rise to the Afrikaner with its Anglo-European descent (Attlee, 1947). Simultaneously, the indigenous expansion of Black Africans to the southern tip of the African continent resulted in approximately 80% of the entire SA population being neither culturally, linguistically, nor genetically homogenous (Van der Merwe et al., 2012).

Additional genetic lineages were introduced by laborers arriving from south Asia. Their arrival resulted in admixture between various groups already residing in SA, including the indigenous African people such as the Khoikhoi, the San, and the African Xhosa tribe (Attlee, 1947). These groups were eventually absorbed into the mixed ancestry group (Oosthuizen et al., 2021). Finally, the last major grouping (Asian population) originated from admixture of individuals from mainland India, neighboring countries such as Bangladesh, and the Mixed Ancestry population of SA (Vishnu and Morrell, 1991). Therefore, the modern-day Asian (specifically the SA Indian) population comprises mostly of people who migrated from mainland India to SA over 300 years, with admixture involving countries from Eurasia and Africa (Mesthrie, 2006; Isaacs et al., 2013). As our genomes reflect a record of historical events, so too does the genetic diversity in the field of hereditary breast and ovarian cancer (HBOC) reflect the complexity of the SA population (Van der Merwe et al., 2020; Combrink et al., 2021; Oosthuizen et al., 2021).

Patients with likely- to pathogenic germline variants in these high-risk genes have an increased predisposition to develop BC and/or ovarian cancer (OVC) throughout their lifetime. According to global statistics, the cumulative risk for *BRCA1* and *BRCA2* mutation carriers to develop BC before 80 years of age is 40%–87% and 27%–84%, respectively. The associated risk for OVC varies from 16%–68% and 11%–30%, respectively (Kuchenbaecker et al., 2017). The etiology related to hereditary BC and OVC in SA derived great benefit from population-based genetic research (Reeves et al., 2004; Agenbag, 2005; Van der Merwe and van Rensburg, 2009; Sluiter and Van Rensburg, 2011; Van der Merwe et al., 2012; Peter, 2014; Chen, 2015; Francies et al., 2015; Combrink, 2016; Moeti, 2016; Oosthuizen, 2016; Van der Merwe et al., 2020; Combrink et al., 2021; Mampunye et al., 2021; Oosthuizen et al., 2021), resulting in the identification of five founder variants representative of three of the four major ethnic groups in the country (*BRCA1* c.1374del [rs397508862], *BRCA1* c.2641G>T [rs39750888] (Reeves et al., 2004); *BRCA2* c.7934del [rs80359688] (Van der Merwe and van Rensburg, 2009); *BRCA2* c.5771_5774del [80359535] (Van der Merwe et al., 2012); *BRCA2* c.582G>A [rs80358810] (Oosthuizen et al., 2021)).

The SA studies performed to date reflect substantial variation in the yield of actionable (likely-pathogenic and pathogenic) germline variants identified in the country. As only 4.5% of the SA total budget is allocated to healthcare expenditure, it burdens an already stressed public sector to seek cost-effective alternatives for routine diagnostic testing of familial breast and ovarian cancer patients. This single-institution public sector study aimed to determine the range and positive mutation percentage of *BRCA1* and *BRCA2* actionable variants in an unselected large cohort of BC and OVC patients. These patients were screened using various technologies ranging from targeted genotyping to comprehensive screening, employing both older and new technologies. Our results prompted us to contemplate the most appropriate workflow for SA state-owned pathology laboratories to provide cost-effective genetic assessment in a financially constraint health sector.

## 2 Materials and Methods

### 2.1 Study Population

**FIGURE 1:**
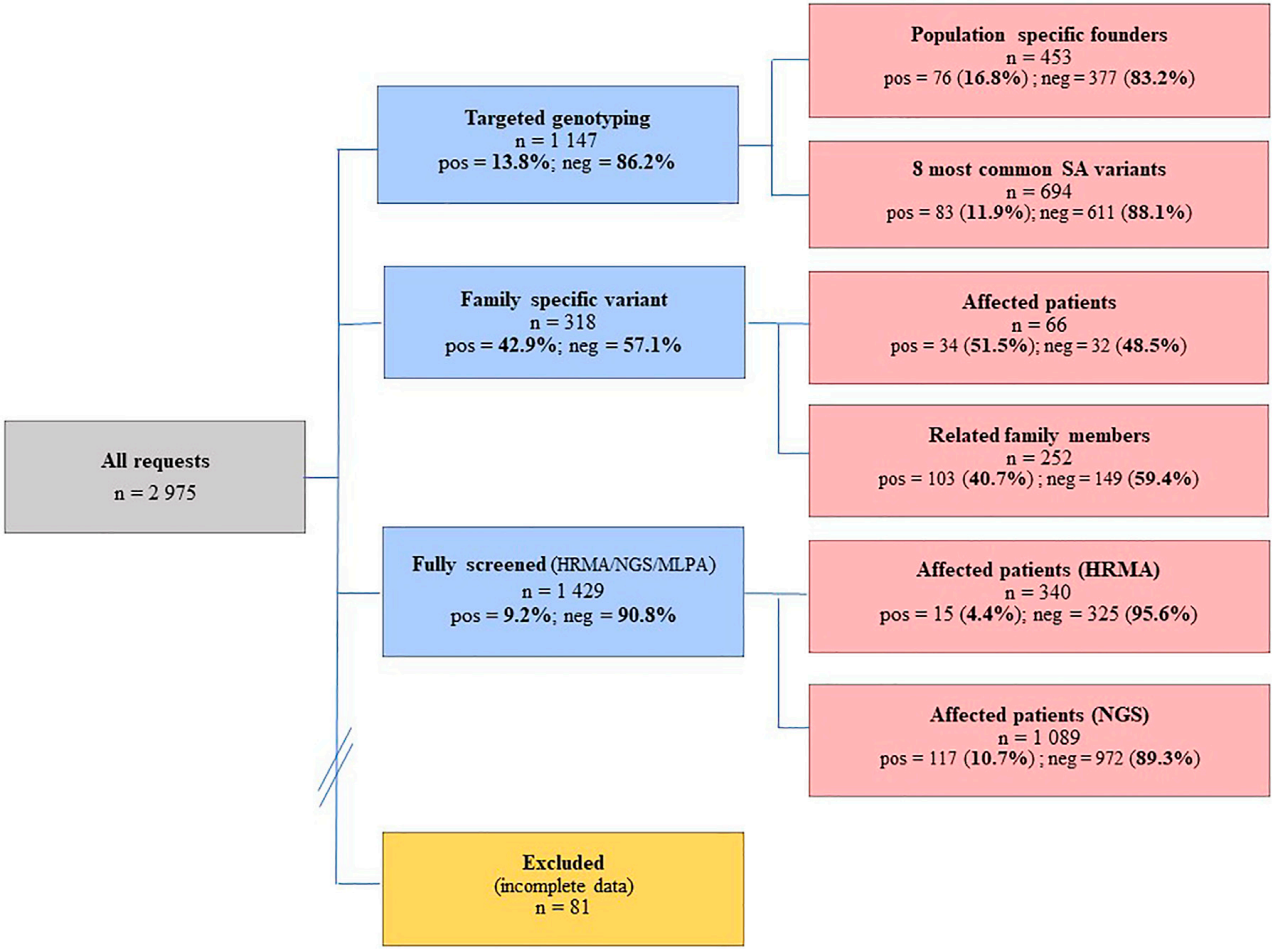
Individuals screened at the NHLS Human Genetics laboratory in Bloemfontein between 1998 and 2020, using a combination of investigative methods ranging from targeted genotyping to comprehensive screening. The numbers screened together with the success rates are indicated. Pos, positives; neg, negatives; HRMA, high resolution melting analysis; NGS, next-generation sequencing; MLPA, multiplex ligation-dependent probe amplification.

A total of 2,975 BC and/or OVC patients were consulted at the National Health Laboratory Service (NHLS) Human Genetics Laboratory in Bloemfontein between 01/01/1998 and 31/12/2020, of whom 81 patients had incomplete data and were excluded from the analysis. The remaining 2,894 BC and/or OVC patients (2,733 females and 161 males) were examined by targeted genotyping or comprehensive screening of *BRCA1/2* (Figure 1). These BC and/or OVC patients attended their closest genetic clinic at a regional or provincial hospital, where they were referred for diagnostic genetic testing through their local genetic counselor or attending physician. Indications for testing included BC diagnosed at age 45 years or less or a significant family history (at least one first-degree family member with premenopausal BC or OVC, or multiple second-degree family members with premenopausal BC and/or OVC, or males with BC at any age).

Ethnicity was determined by patients’ self-identification at the time of consultation. The major ethnic group classifications have sub-classifications, some contextualized specifically to the SA demographic profile (Table 1). The majority (*n* = 975) was Caucasian, followed by the Black African (*n* = 753) and mixed ancestry groups (*n* = 669), with the Asian group being the minority (*n* = 297). The ethnicities for the remainder of patients (*n* = 200) were either not indicated or unknown. The distribution of the patients across the various ethnic groups reflected the demographic profile of the breast clinic only and did not reflect BC incidence within each of these groups. Additionally, patient reports generated over the years were retrospectively analyzed to collect information on epidemiological characteristics, which specifically focused on 1) presence of a family history of BC and/or OVC; 2) age at onset; 3) stage of the disease; 4) ethnicity; and 5) mutation status.

**TABLE 1:** Self-identified ethnicity of consecutive BC and/or OVC patients included in the study (*n* = 2,896).

Major ethnic classification	Sub-ethnic classification	Count
Asian	South African Indian, Malay, Pakistani, Chinese, Japanese, Bengali, Mongolian	297
Black African	All Black African nationalities	753
Caucasian	South African Afrikaner, British, and European descent	975
Mixed ancestry	A South African subgroup of people that comprise a mixture of any of the abovementioned ethnic classifications	669
Unknown	Not specified	200

Majority of individuals included in this cohort were diagnosed with BC and/or OVC. All the individuals had received pre- and post-test counseling at their respective referral centers. Information regarding personal and family history, as well as written informed consent for DNA testing, were obtained. The cohort included patients with a positive family history (two or more affected family members) for BC and/or OVC, with most representing low-risk patients who had no prior knowledge of a family history of either condition but were diagnosed at an early age of onset (<40 years). The Health Sciences Research Ethics Committee (HSREC) of the University of the Free State in Bloemfontein, SA, approved all study protocols submitted since 1995 (ETOVS 31/95; ECUFS 31B/95; ECUFS 31C/95; ETOVS 49/06; ETOVS 65/08; ECUFS 107/2014; ECUFS 108/2014; UFS-HSD2019/1835/2910 and UFS-HSD2020/0194/3006). The NHLS permitted the use of the data (reference PR2110611).

### 2.2 Laboratory Methods

DNA was extracted from peripheral blood using a standard extraction method. The concentration and purity were determined using spectrophotometry. Targeted genotyping for the founder and recurrent actionable variants was performed using the LightCycler^®^ 480 Genotyping Master Mix (Roche Diagnostics, Germany) on the LightCycler^®^ 480 II real-time instrument (Roche Diagnostics, Germany). These assays employ hybridization and simple probe technology described by Oosthuizen (Oosthuizen, 2016). Targeted genotyping was performed using a standard real-time PCR regime, followed by melt curve analysis. Conventional mutation screening for single nucleotide variants (SNVs) and smaller indels was initially performed for a subset of patients described previously (Combrink, 2016; Oosthuizen, 2016). This approach involved a combination of older technology-based techniques including High-Resolution Melt Analysis (HRMA), the protein truncation test (PTT) and Sanger sequencing (Van der Merwe et al., 2020). A total of 340 patients were screened using a combination of these older techniques (Figure 1).

Individuals screened using the conventional approach were subjected to copy number variants (CNVs) analysis using Multiplex Ligation-dependent Probe Amplification (MLPA). The assays used included the SALSA^®^ MLPA^®^ P002-C1 and SALSA^®^ MLPA^®^ P002-D1 for *BRCA1*, with SALSA^®^ MLPA^®^ P045-B3 used for *BRCA2* (MRC-Holland, Amsterdam, Netherlands). The products were run together with a size standard on an ABI 3130XL Genetic analyzer (Applied Biosystems, Carlsbad, CA, United States). MLPA-positive results, especially in the case of single exon deletions, were corroborated using an alternative confirmation assay for each of the genes, namely SALSA^®^ MLPA^®^ P087-C1 for *BRCA1* and SALSA^®^ MLPA^®^ P077-A3 for *BRCA2*. All the data were analyzed using GeneMarker^®^ software version 2.6.4 (SoftGenetics, LLC, State College, PA, United States). The CNVs were named according to the Human Genome Variation Society (http://www.HGVS.org/varnomen) guidelines and classified using the adapted recommendations of the American Society of Medical Genetics and Genomics (ACMG) for the interpretation and reporting of single-gene CNVs (Brandt et al., 2020).

Once introduced, next-generation sequencing (NGS) was performed to screen the remainder of samples (*n* = 1,089), using the Oncomine™ BRCA Research Assay (Life Technologies, Carlsbad, CA, United States). The primer pools targeted both genes’ entire coding region and splice-site junctions. Multiplexed primer pools were used to construct the amplicon library using PCR-based targeted amplification. Sequencing was performed on the Ion Proton and S5 Platforms (Life Technologies, Carlsbad, CA, United States), and the Ion Reporter™ Software was used to filter out artifacts and annotate the variants. Raw signal data were analyzed as described by Van der Merwe et al. (2020). The average read-depths obtained were 581×, ranging between 148 and 1,965×. Using the depth per read, quartile statistics were applied to calculate average depth distribution around the mean across the NGS samples. Samples that were located within the 2nd and 3rd quartiles were selected to construct a CNV baseline with the Ion Reporter CNV VCIB 4.0.0.1 algorithm. CNV detection was performed using an algorithm that normalized depth coverage across amplicons to predict the copy number or ploidy states. The computed baseline included a minimum of 100 control samples (each with an average of 24 million bases called and a read count of 215,000), using regions with known ploidy states (https://assets.thermofisher.com/TFS-Assets/LSG/brochures/CNV-Detection-by-Ion.pdf). MLPA was performed to confirm all CNVs detected using NGS. Novel or complex sequence variants were confirmed by means of Sanger DNA sequencing (ABI Prism BigDye^®^ Terminator v3.1 cycle sequencing kit, Foster City, CA, United States), using an Applied Biosystems 3130 automated sequencer (Life Technologies, Foster City, CA, United States).

The clinical significance of variants was determined based on the American College of Medical Genetics guidelines (ACMG, classification confirmed on 02/12/2021) (Richards et al., 2015) and evaluated from freely accessible public databases such as ClinVar and the genomic search engine VarSome (Kopanos et al., 2019). The variant nomenclature was used according to Human Genome Variation Society (HGVS) recommendations (http://www.hgvs.org/rec.html). *BRCA1/2* variants were numbered and annotated using the National Center for Biotechnology Information (NCBI) chromosomes and transcript reference sequences (NC_000017.11, NM_007294.4 and NC_000013.11, NM_000059.3), respectively. To prevent potential biases between the different laboratory techniques utilized for mutation screening throughout the years, all potential nucleotide changes were confirmed using Sanger sequencing. The analyses were confirmed using the same mutation detection databases and reference sequences.

## 3 Results

### 3.1 Composition of Cohort

The influence of various SA founder variants is reflected in the relatively high mutation-positive rates observed for targeted genotyping (Figure 1), with 16.8% observed using population-directed targeted genotyping (*n* = 453). Although the initial screening was based on the individual’s ethnicity, the positive mutation rate decreased when cases were screened using a broader approach that included all of the commonly occurring SA variants (11.9%, *n* = 694). Of the 1,429 patients comprehensively screened, 137 (9.2%) carried a likely- to pathogenic *BRCA1/2* variant. Although the majority represented SNV changes consisting predominantly of substitutions and deletions, eight CNVs were identified. Regarding the SA mutation spectrum, the Afrikaner founder variant *BRCA2* c.7934del (rs80359688) was the most common, followed by the Black African founder variant *BRCA2* c.5771_5774del (rs80359535).

An inherited susceptibility was confirmed in 51.5% of affected family members tested (*n* = 66), that carried the actionable variant segregating in the family (Figure 1). A relatively low number of unaffected family members (*n* = 252) were genotyped for various family-specific variants. The positive mutation rate for this group was high (40.7%). By knowing their mutation status, these patients were included in various cancer screening programs to facilitate earlier detection and a potentially better prognosis in case of a cancer diagnosis.

### 3.2 Epidemiology

The epidemiological data were analyzed according to the five variables highlighted, namely a family history of BC and/or OVC, age at onset, stage of the disease, ethnicity and mutation status. Regarding the age at onset, the majority of patients tested fell in the 40–49 age group (collectively 27.7%), followed by the 50–59 age group (21.2%) (Table 2). These two age intervals also delivered the highest percentage mutation-positive results compared to the other age groups, namely 4.5% and 3.8%, respectively. Only three (0.5%) of the patients identified with a *BRCA1/2* actionable variant in the 20–29 age group were observed. These positive patients represented approximately one-third of patients tested in this age group. These patients either represented high-risk BC/OVC families or were diagnosed with aggressive disease early and were therefore genetically screened (Table 2).

**TABLE 2:** Comparison between the number of mutation-positive versus mutation-negative patients (reflected in percentages) observed per ten-year intervals.

Age group	Mutation negative (*n* = 2,413)	Mutation positive (*n* = 481)	Total (%)
0–19 (*n* = 3)	0.10	0.00	0.10
20–29 (*n* = 62)	1.66	0.48	2.14
30–39 (*n* = 480)	13.96	2.63	16.59
40–49 (*n* = 800)	23.15	4.49	27.64
50–59 (*n* = 616)	17.45	3.84	21.29
60–69 (*n* = 467)	13.41	2.73	16.14
70–79 (*n* = 278)	8.05	1.55	9.61
80–89 (*n* = 108)	3.25	0.48	3.73
90+ (*n* = 21)	0.59	0.14	0.73
Unknown (*n* = 59)	1.76	0.28	2.04
Total (%)	83.38	16.62	100.00

Of the 481 mutation carriers identified, the majority were Caucasian. However, the data for this grouping were skewed due to the translational research performed before 1998, revealing the presence of three common founder variants in the Caucasian sub-category Afrikaner group (Table 1). This research initially involved mostly Afrikaner patients with a positive family history of BC and/or OVC being screened. This group served as the ideal research group due to proven extended high linkage disequilibrium with various founder effects reported. This SA group is considered a fruitful “hunting ground” for pathogenic variants associated with disease (Hall et al., 2002; Van der Merwe et al., 2012). The presence of these *BRCA1/2* founder variants increased the mutation positivity rate to a remarkable 8.12%, delivering the highest positivity rate for Caucasians, namely 24.1%. The mutation positivity rate is an indicator used as a proxy for the relative percentage of patients that tested positive out of the total sample population (Table 3).

**TABLE 3:** Illustration of the mutation detection and positivity rate per major population group (reflected in percentages) observed for the major groups.

Ethnic group	Mutation positive (*n* = 481)	Mutation negative (*n* = 2,413) (%)	Mutation positivity rate (%)	Total (%)
Asian (*n* = 297)	1.62	8.64	15.8	10.26
Black African (*n* = 753)	3.77	22.25	14.5	26.02
Caucasian (*n* = 975)	8.12	25.57	24.1	33.69
Mixed ancestry (*n* = 669)	2.70	20.42	11.7	23.12
Unknown (*n* = 200)	0.41	6.50	6.0	6.91
Total (%)	16.62	83.38	16.6	100.00

Information regarding the presence or absence of a family history of BC and/or OVC was available for most patients (Supplementary Table S1), with 5.8% designated as unknown (*n* = 141). Patients adopted as children contributed to the unknown category, as they had no prior family information. More than 80% of patients (*n* = 392) carrying an actionable *BRCA1/2* variant reported family members affected with BC and other cancer types. Although the information regarding family structure varied from being limited (lacking maternal or paternal lineages and ages at diagnoses) to extensive (three-generation pedigrees with both paternal and maternal lineages indicated), the percentage highlights the consistent importance of determining the family history as an effective selection criterion for *BRCA1/2* genetic testing. Despite reporting a positive family history of BC and/OVC, no actionable variants were detected for 59.9% (*n* = 1,445) of patients screened (Supplementary Table S1).

As the African continent has previously been associated with more aggressive breast disease and higher mortality rates due to late-stage presentation, the stage at diagnosis was compared between the ethnicities for 455 patients for whom the relevant information was available (Supplementary Table S2). The stage of disease at diagnosis for two groups (the Caucasians and individuals of mixed ancestry) was similar, with most patients diagnosed with Stage 2 disease. These two groups had the highest percentage of Stage 1 BC (10.6%), indicating increased community awareness and successful BC screening programs in the public health sector. These groups also exhibited a low number of patients diagnosed with Stage 4 disease (8.5% and 6.1%, respectively; Supplementary Table S2). This pattern was similar to that for the Black African group, except Stage 3 disease being the most prevalent. This finding still hints towards a later stage at presentation. However, it has improved significantly as a mere 7.6% of patients had Stage 4 disease at diagnosis. The Asian population of SA was the most alarming of the four ethnic groups due to the high percentage of patients diagnosed with Stage 4 disease (16.2%). However, this percentage could have been skewed due to the small sample size of this particular group (*n* = 37).

Patients affected with BC and/or OVC were divided according to unilateral and bilateral disease related to the presence or absence of a *BRCA1/2* variant (Supplementary Table S3). The majority of patients presented with unilateral BC, with only 220 cases affected with bilateral disease and a further 91 affected with OVC. A small number of patients (*n* = 62) were also diagnosed with a secondary cancer type not specified here (Supplementary Table S3). The mutation-positive rates in this cohort varied between the two BC groups, with each group exhibiting similar success rates. For the OVC cases, the detection rate was considerably higher (21.9%), as 20 patients in total carried *BRCA1/2* actionable variants (9 in *BRCA1* and 11 in *BRCA2*).

### 3.3 Mutation Spectrum

Apart from targeted genotyping, mutation screening of 1,429 patients revealed a wide range of variants across the SA population groups. The data were generated from 340 patients screened using older mutation screening techniques, with a further 1,089 assessed by NGS. The data of the two sets were incorporated and are presented in Table 4. A total of 132 (9.2%) patients representing 73 likely- to pathogenic variants were identified (*n* = 117 for NGS and *n* = 15 using older technologies), with 57.6% (76/132) representing *BRCA2*. Twenty-two of these actionable variants were classified as splice-site variants, mainly located in the intronic splice site boundaries. Various CNVs were detected, ranging from single exon to complete gene deletions. These CNVs have been previously described by Van der Merwe et al. (2020). Across the genes, NGS detected 344 variants, with only 14% present in a homozygous state. Unique variation in the SA population resulted in 196 variants identified with a minor allele frequency (MAF) below 0.01, with 47 consequently being classified as variants of unknown clinical significance (VUS), predominantly in *BRCA2*. Of these 47 variants, 30 were completely novel and not detected in international databases used for variant interpretation (Table 4).

**TABLE 4:** Summary of variants detected during comprehensive screening of 1,429 patients.

Variant category	Patients (*n*)	Variants (*n*)	Homozygous variants (*n*)	Heterozygous variants (*n*)
*BRCA1* actionable variants	55	34	0	34
*BRCA2* actionable variants	76	39	0	39
Total # of *BRCA1* variants	na	146	22	124
Total # of *BRCA2* variants	na	198	26	172
Total # of VUSes in *BRCA1*	30	17	0	17
Total # of VUSes in *BRCA2*	96	30	0	30
*BRCA1* variants with MAF <0.01	na	84	1	83
*BRCA2* variants with MAF <0.01	na	112	0	112
Splice-site variants in *BRCA1*	na	11	0	11
Splice-site variants in *BRCA2*	na	11	0	11
Novel variants in *BRCA1*	na	13	1	12
Novel variants in *BRCA2*	na	17	0	17
Copy number variants in *BRCA1*	13	6	1	6
Copy number variants in *BRCA2*	2	2	0	2

VUS, variant of unknown clinical significance; na, not applicable.

The mutation positivity rates for the main SA ethnicities varied, with 9.2% reported for the Black African group (44/479), 6.6% for the Asians (12/180), 18.1% for the Caucasians (25/138), 13.2% for the mixed ancestry group (22/167), with 11.8% allocated to the group of unknown ethnicity (14/118). From the 59 actionable variants detected across the populations using NGS, 23 were detected in the Black African group, 10 in the Asian group, 12 in the Caucasian group, 13 in the mixed ancestry group, with 12 falling into the group of unknown ethnicity. Only seven of the 59 variants were detected in two separate populations, with a single pathogenic variant (*BRCA2* c.582G>A [rs80358810]) observed across all four main ethnic groups. However, the remainder of the actionable variants (51/59) were restricted to a single population group. Recurrence of these likely- to pathogenic variants was low in the NGS cohort with 66.1% (39/59) observed for a single patient. Another 18.6% of the variants (11/59) were detected twice, followed by 6.8% (4/59) identified in three patients each. A small percentage (8.5%) was common and represented the three most common founder variants, namely *BRCA2* c.5771_5774del (detected in eight patients), *BRCA2* c.582G>A (10 patients) and *BRCA2* c.7934del (detected 17 times in the NGS cohort alone).

### 3.4 Genomic Consequence

**FIGURE 2:**
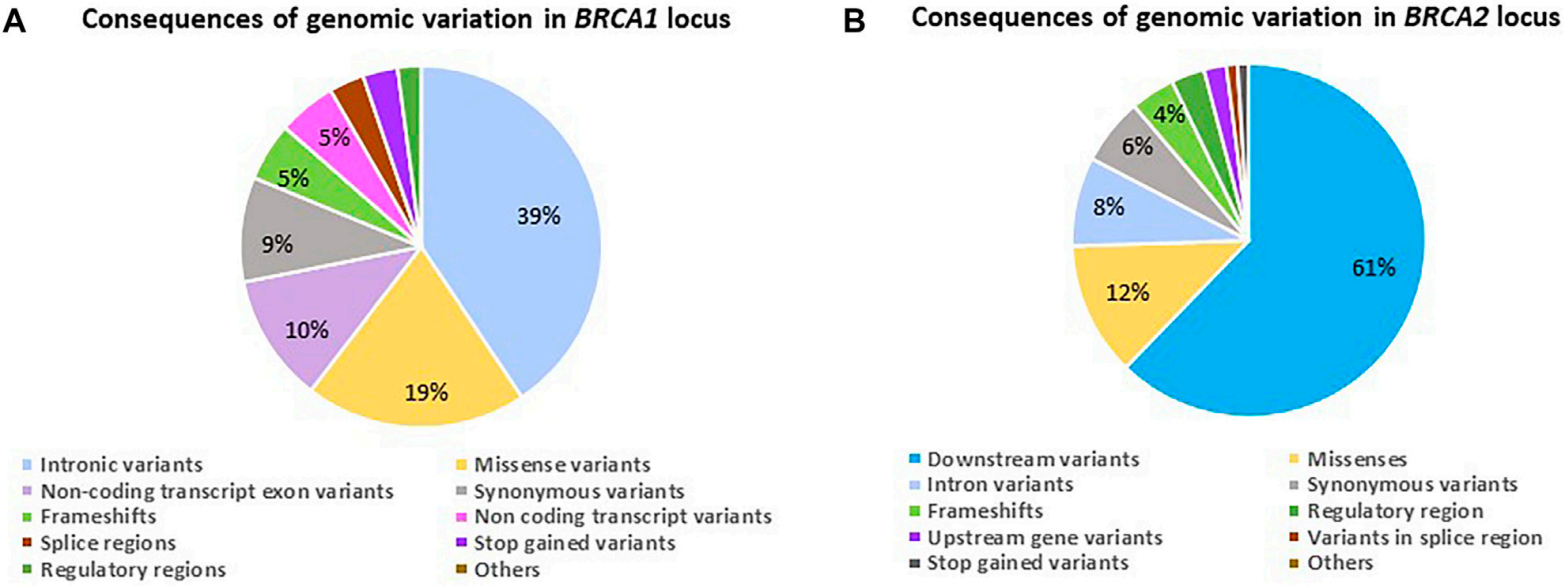
Diagrams indicating the genomic variant frequencies for the *BRCA1* and *BRCA2* loci amongst 1,089 patients screened using NGS. **(A)** The consequence ratios relative to variant frequencies observed for the *BRCA1* locus. **(B)** The consequence ratios relative to variant frequencies observed for the *BRCA2* locus.

When investigating the consequences of the variation observed, it varied for the two genes (Figures 2A,B). The genomic variant frequencies and associated consequences were only based on the NGS data. Although the bulk of variation for *BRCA1* was represented by non-coding transcript variants primarily present in the intronic regions (Figure 2A), the consequence of these variants has the potential to affect both overlapping genes, namely *BRCA1* (NM_000294.4 [43,044 295–43,125 364] and *Homo sapiens* Rho family GTPase 2 (*RND2*—NM_005440.5 [43,025 231–43,032 041]), involving a total of eight overlapping transcripts and five regulatory features. For *BRCA2*, the variation was present in the form of downstream changes observed in the 3′ untranslated region (Figure 2B). In comparison, the genomic variation observed in this section of chromosome 13 has the potential to affect three overlapping genes, namely *BRCA2* (NM_000059.4 [32,315 508–32,400 268]), *Homo sapiens* zygote arrest 1 like (*ZAR1L*—NM_001136571.2 [32,303 699–32,315 363]) and NEDD4 Binding Protein 2 Like 2 (*N4BP2L2*—NM_001,387,001.1 [32,432 485–32,538 795]), encompassing 28 overlapping transcripts and four regulatory features.

**FIGURE 3:**
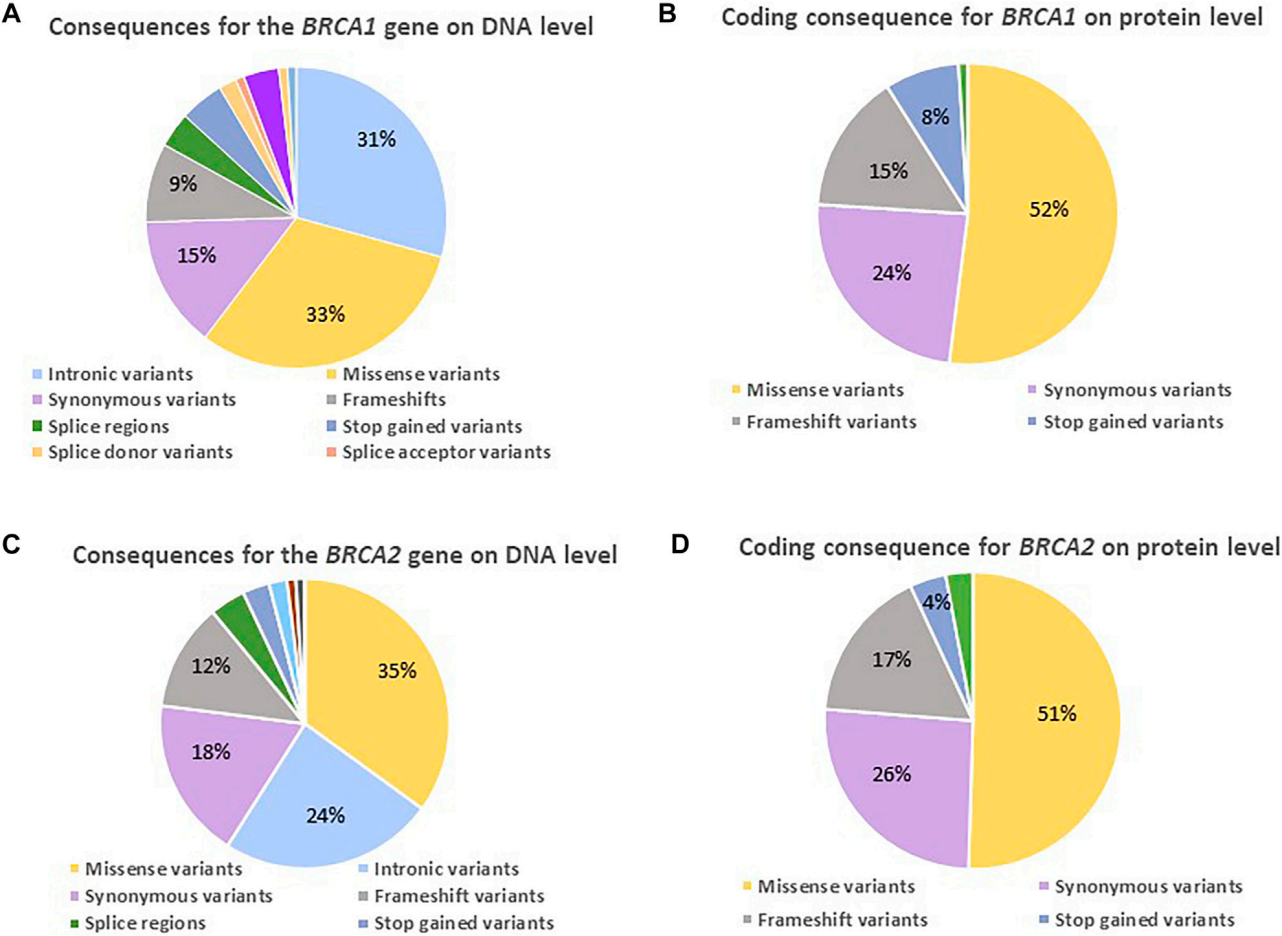
Diagrams indicating the variants detected for the two familial BC genes on DNA and protein level amongst 1,089 BC and/or OVC patients screened using NGS. **(A)** The consequence ratios (in percentages) predicted for *BRCA1* on a DNA level. **(B)** The consequence ratios predicted for *BRCA1* on a protein level. **(C)** The consequence ratios predicted for *BRCA2* on a DNA level. **(D)** The consequence ratios predicted for *BRCA2* on a protein level.

On DNA level, only a small percentage of the observed *BRCA1* changes represented variants potentially having an impact, namely frameshift (9%), splice- (3%), and stop-gained variants (5%) (Figure 3A). Their contribution increased on protein level (Figure 3B), directly impacting the protein and consequently efficient DNA repair, as 23% resulted in a prematurely truncated peptide (15% frameshift and 8% stop-gained variants) together with 5% missense variants. The majority of the actionable variants were detected in *BRCA1* exon 10. Approximately half of the variants observed at protein level represented missense variants, of which 14/55 changes were classified as VUSes. The missense variants were distributed throughout the gene, with the majority located outside of functional or disordered domains (Table 4).

Although there was an enrichment of actionable *BRCA2* variants in our populations (Table 4), the profile regarding the composition and impact of the variants on DNA and protein level corresponded between the two genes (Figures 3A–D), with the exception that the majority missenses of unknown significance were detected in a functional domain or disordered region. *BRCA2* did reveal a smaller margin of intronic variants (Figure 3C), as the bulk of non-coding variation was in the 3′ untranslated region. The high percentage of missense variants in the heterozygous form illustrated the unique composition of the SA populations, contributing to the rate of VUSes identified due to a lack of African data in the international context (Table 4).

## 4 Discussion

### 4.1 Targeted Genotyping

Translational research performed at the University of Free State in collaboration with Professor L Jansen van Rensburg at the University of Pretoria resulted in the identification of the first two recurrent SA pathogenic variants in *BRCA1*, namely c.1374del (legacy name 1493delC) and c.2641G>T (legacy name E881X) observed for the Afrikaner population. Based on this research, *BRCA1* c.2641G>T was the first SA pathogenic variant to receive founder status, proven by haplotype analysis that indicated a single mutational event (Reeves et al., 2004). This variant was soon followed with founder status for *BRCA1* c.1374del and *BRCA2* c.7934del (legacy 8162delG). These three founder variants (with *BRCA2* c.7934del being the most common) were initially restricted to familial BC families with an Afrikaner heritage. Their founder status was corroborated with genealogical evidence dating back over 13 generations. For each variant, a single founding couple was identified based on genealogical findings traced back to France (*BRCA1* c.2641G>T), the Netherlands (*BRCA2* c.7934del) and Belgium (*BRCA1* c.1374del) (Reeves et al., 2004; Van der Merwe and van Rensburg, 2009).

The research resulted in the first diagnostic test (entailing screening for the three Afrikaner founder variants) offered to patients in the public sector in 1998. Diagnostic testing was initially restricted to Afrikaner individuals as translational research was performed in parallel to identify pathogenic variants in the other SA population groups (Agenbag, 2005; Sluiter and Van Rensburg, 2011; Van der Merwe et al., 2012; Peter, 2014; Chen, 2015; Combrink, 2016; Moeti, 2016; Oosthuizen, 2016). With time, various non-Afrikaner recurrent variants had been identified that were proven to represent recurrent variants, which include *BRCA2* c.5771_5774del (legacy 5999del4) and *BRCA2* c.582G>A, both Black African founder variants (Van der Merwe et al., 2012; Oosthuizen et al., 2021).

As the demand for diagnostic testing increased over the years, patients were collectively tested for the eight most commonly occurring SA variants, irrespective of ethnicity. This mutation set included the three Ashkenazi Jewish/European founder variants based on the African Lemba tribe’s affinity with the Ashkenazi Jews (Spurdle and Jenkins, 1996) and the SA Indian community to mainland India and Europe (Combrink, 2016). This targeted genotyping assay served as a first-tier test for all patients that proved justified, because with time, the admixture of the SA populations became evident based on the genotyping results. The founder variants were no longer restricted to a single ethnicity but were shared among groups. This situation was expected, as recently illustrated by the findings of Hollfelder et al. (2020). With the use of five million genome-wide markers, 77 Afrikaner individuals were genotyped. Although the bulk of the genetic information represented Europeans, the study indicated a contribution of 1.7% from South Asia or India, 1.3% from the Khoikhoi and the San and 0.8% representing West and East Africa (Hollfelder et al., 2020).

The results obtained from this series indicate that not all variants currently included in the first-tier genotyping assay are worthy of inclusion due to their low prevalence. This applies to the three Ashkenazi Jewish founder/European variants and *BRCA1* c.1374del, the rarest Afrikaner founder variant. The maximal financial benefit will be obtained once the first-tier assay has been re-designed to include the most commonly occurring variants in the SA population. It will aid the financially depleted healthcare system by first screening patients for the most common *BRCA1/2* variants in the population before advancing to more costly NGS (Oosthuizen et al., 2021).

### 4.2 Epidemiology

The epidemiology data highlighted three important aspects to be considered for the SA population. The first entailed the relatively high percentage of young individuals (20–29 years) identified with an actionable variant (0.5%, Table 2). Fifty-six percent of the group (*n* = 62) was unaffected and was referred for carrier testing only. For 22.8% of these individuals, targeted genotyping revealed a positive test result. By knowing their mutation status so early in life, these women were empowered by informed screening decisions and potentially alternative management options. Maximum benefit was therefore gained to reduce their risk of developing cancer types associated with *BRCA1/2* pathogenic variants. Asymptomatic young mutation carriers can reduce their risk of BC mortality by being regularly monitored and taking up interventions such as preventive surgery and/or chemoprevention (McCarthy and Armstrong, 2014).

The remainder (44%) of the 20–29 group was affected with early-onset BC (*n* = 27), with ages at onset ranging from 20 to 29 (average 26.7) years. The patients represented the Black African (*n* = 15), mixed ancestry (*n* = 5) and Caucasian (*n* = 6) ethnicities. Twenty-two percent (*n* = 6) of these patients received a positive test result (average age at onset 22.5 years), with the majority self-identified as Black African females. Half of these mutation-positive patients presented with a family history of BC and other cancer types, such as prostate cancer, melanoma and pancreatic cancer. Although these females were diagnosed at such a young age, knowing their *BRCA1/2* status promoted informed decision-making regarding treatment options and potential surgery. With a familial *BRCA* variant identified so early, cascade testing of related family members can proceed to assist with risk-reducing interventions themselves.

According to the latest clinical guidelines released for BC control and management by the SA Department of Health in 2018 (National Department of Health of the Republic of South Africa, 2018), all these patients qualified for genetic services, yet the numbers genetically screened are meager (Table 2). With a positive detection rate between 6% and 24%, attention should be drawn to genetic screening, as the benefits would outweigh the financial costs by far (Table 3).

The epidemiological evidence highlighted the ever-important value of a family history as a potential predictor of a positive test result (Supplementary Table S1), as 90% of patients with an actionable *BRCA1/2* variant reported family members affected with BC and other cancer types. Identifying an actionable *BRCA1/2* variant in an index acts as a key to the entire family. Once a mutation carrier has been identified, the benefits of genetic testing can be magnified through sharing positive test results with at-risk related family members. Doing so will ensure that they benefit from this information and secure their access to predictive testing according to the latest guidelines. This approach is of tremendous importance from a public health perspective, pushing for improved survival and quality of life through earlier detection and optimal management.

Conley et al. (2020) recently investigated the family disclosure patterns of *BRCA* genetic test results among young Black women with invasive BC in the United States of America. The study examined whether a disclosure to relatives was made, and if so, to whom it was disclosed. The authors reported that the most significant benefit of genetic testing, namely to inform family members of a hereditary predisposition, is not being realized in Black American families. Their findings revealed a reluctance of mutation-positive patients to disclose a positive test result even to their daughters (Conley et al., 2020).

The reluctance to share genetic test results with family members is also evident in SA. Here it is not restricted to the Black African population but clearly occurs among all ethnic groups in SA apart from the Caucasians. Despite 481 individuals receiving a positive test result, a mere 318 predictive or carrier tests had been performed over 24 years. The majority was performed before 2003 as a post-research initiative involving Afrikaner families. From the epidemiological results, it appeared that Caucasians tended to value and discussed the outcome of genetic testing to a greater extent than the other main ethnic groups (Table 1). This trend was also noted by Armstrong et al. (2003), who found that women pursuing *BRCA1/2* genetic testing in the United States were significantly more likely to be Caucasian.

This issue has been investigated internationally and although the public attitudes towards genetic testing for the risk of diseases, including cancer, are generally positive, various studies highlighted areas of concern. These include factors such as language barriers, fear of discrimination against those with a genetic predisposition for illness (Haga et al., 2013), being labeled as an individual or family with “good” or “bad” genes (Henneman et al., 2013) and fatalistic views of cancer (Allford et al., 2014). In SA, Schoeman et al. (2013) previously reported a low level of awareness regarding genetic testing for BC and other cancer types. Despite genetic testing being available since 1998, recognizing the value of a predictive test remains low. SA, therefore, needs to investigate innovative approaches to increase awareness among patients and communities, starting with related family members of mutation carriers. As the workforce of genetic providers is extremely low both in SA and globally, the international genetics profession has attempted to adapt to the situation by offering genetic counseling via alternative new methods, including service delivery via telephone, telegenetics and group genetic counseling. Although a face-to-face consultation is always the ideal model to strive for, innovative service delivery models such as group counseling can improve access and contribute to community awareness.

In the paper by Mampunye et al. (2021), one such innovation was described for SA, which investigated the clinical value of rapid point-of-care (POC) genetic testing performed in combination with genetic counseling. As the ParaDNA workflow involves an integrated system from sample collection to report generation, prospective validation using a non-invasive cheek swab or saliva as input DNA is warranted (Mampunye et al., 2021). This approach would be ideal for use as a first-tier test performed by trained healthcare providers in parallel with genetic counseling in rural primary health clinics. This will provide the opportunity to improve the care process by delivering on-demand psychosocial support directly to the patient and indirectly to the community where it is needed. This approach could have many benefits: 1) reducing the lack of early healthcare-seeking due to limited financial resources and transport difficulties; 2) increasing community knowledge and cancer awareness resulting in less stigmatization; 3) creating an opportunity to explain the benefits of knowing your *BRCA1/2* genetic status for evidence-based cancer treatment options; and 4) highlighting the benefits of testing for at-risk family members in the case of a positive test result, thereby increasing preventative actions and early detection (Mampunye et al., 2021).

Unfortunately, the burden of BC-related death is ever increasing due to persisting misconceptions surrounding the disease and various other socio-economic factors such as poverty, cultural and religious beliefs (Van der Merwe et al., 2020; Mampunye et al., 2021; Oosthuizen et al., 2021). Despite the efforts of the SA Department of Health’s BC development plan establishing multiple Regional Breast Units (RBUs, 28 distributed throughout the various provinces) at primary and secondary state hospitals, and 14 additional Specialized Breast Units (SBUs) located in mostly tertiary hospitals (National Department of Health of the Republic of South Africa, 2018), the uptake of breast screening remains low in women 30 years and older (Phaswana-Mafuya and Peltzer, 2018). The authors of this large study (*n* = 10,831) estimated a low prevalence of BC screening similar to that obtained in a 2008 survey involving older SA adults, 50 years and older (Peltzer and Phaswana-Mafuya, 2014). Their estimation corresponded with the prevalence observed in international low-income countries such as Thailand (Mukem et al., 2016) and Turkey (Sözmen et al., 2016), but was lower than that of Brazil (Theme Filha et al., 2016). For SA to advance in this battle and attack BC energetically and effectively, we have to invest in community-health educational out-reaches performed in parallel with highly specialized science to achieve the ultimate goals set for the country.

The epidemiological data finally emphasized the value of screening all OVC patients for actionable variants in *BRCA1/2*, as screening resulted in a mutation-positive rate of 28.2% (Supplementary Table S3). Nine (45.0%) of the 20 OVC mutation carriers exhibited one of the SA founder variants, with the remainder of actionable variants being family-specific. These founder variants could easily have been identified cost-effectively using first-tier targeted genotyping, before proceeding to comprehensive genetic analysis. A high number of these OVC *BRCA1/2* mutation carriers, however, was referred for identification of a rare family-specific variant. Although patients affected with OVC before the age of 60 are included in the national clinical guidelines for the control and management of BC (National Department of Health of the Republic of South Africa, 2018), only 91 OVC patients were received between 1998–2021. This number accounts for 3.9 patients screened per year in the public sector. Currently, the age-standardized incidence rate for OVC in southern Africa is 3.9 per 100,000 (The Council for Medical Schemes, 2019). Based on these statistics, the majority of OVC patients are currently not referred for any *BRCA1/2* screening.

Screening these patients could have a two-fold advantage. International ongoing clinical trials involving poly (ADP-ribose) polymerase (PARP) inhibitors have recently resulted in the approval of various inhibitors by the Food and Drug Administration within the US Department of Health and Human Services for clinical use in specifically epithelial OVC patients (Loizzi et al., 2020). This new therapeutic approach for the management of OVC has been suggested in particular for patients with assessed defects in the homologous recombination DNA repair process such as *BRCA1/2* (Sunada et al., 2018; Elias et al., 2018). By knowing their mutation status, patients will be appropriately selected for this new revolutionary treatment option. Unfortunately, the use of PARP inhibitors is not yet registered by the South African Health Products Regulatory Authority and their use in treatment requires Section 21 approval for the private sector (The Council for Medical Schemes, 2019). It is currently not available for the clinical treatment of patients in the public sector.

### 4.3 Mutation Spectrum

We aimed to describe clinically relevant germline *BRCA1/2* variants and their distribution across ethnicities in the most extensive unselected African series to date for the public sector. The data represent the current status after 23 years of *BRCA* testing within our state laboratory. Although testing commenced with a tiered approach in the form of research-based translational targeted genotyping, the total number of test requests and the number of variants identified soared within the past decade. This could be attributed to a heightened public awareness after the Angelina Jolie revelations in 2013 (Troiano et al., 2017) and the incorporation of NGS into our diagnostic workflow, which enabled a more rapid throughput and resulted in more effective variant discovery.

A total of 481 individuals were identified as high-risk *BRCA1/2* mutation carriers, with 69.6% (*n* = 335) representing patients affected by BC and/or OVC, accounting for 9.2% of patients comprehensively screened (132/1,429). The data revealed an extreme distribution for each gene, with only a few frequent pathogenic variants identified. The majority of variants were extremely rare and primarily family-specific (Table 5). Although the proven founder or recurrent variants for the Ashkenazi Jewish, Afrikaner and Black African/mixed ancestry variants were the most common variants observed in terms of the highest frequencies, they only represented <10% of the variants identified (4/55 for *BRCA1* and 5/57 for *BRCA2*, Table 5).

**TABLE 5:** Actionable *BRCA1/2* variants (likely- to pathogenic) identified for the entire SA cohort (*n* = 2,896).

Variant	Protein	Cancer type in index or family	Exon	#Of families	rs number
***BRCA1***					
NC_000017.11:g.(?_43045584)_(43125327_?)del		BC	1 – 23	1	no rs
NC_000017.11:g.(?_43123946)_(43125327_?)del		BC & OVC	1 – 2	5	no rs
NC_000017.11:g.(?_43104032)_(43106675_?)del		BC & OVC	4 – 6	1	no rs
NC_000017.11:g.(?_43082330)_(43082599_?)dup		BC & OVC	12	2	no rs
NC_000017.11:g.(?_43063781)_(43064034_?)del		BC & OVC	17	3	no rs
NC_000017.11:g.(?_43048992)_(43049260_?)del		BC & OVC	21	1	no rs
NM_007294.4(BRCA1): c.45dup	NP_009225.1: p.Asn16Ter	BC	2	4	rs730881457
NM_007294.4(BRCA1): c.66dup	NP_009225.1: p.Glu23ArgfsTer18	BC	2	5	rs80357783
NM_007294.4(BRCA1): c.68_69del	NP_009225.1: p.Glu23ValfsTer17	BC & Gastric ca	2	8	rs80357914
NM_007294.4(BRCA1): c.71G>C	NP_009225.1: p.Cys24Ser	BC	3	2	no rs
NM_007294.4(BRCA1): c.110C>A	NP_009225.1: p.Thr37Lys	BC & OVC	3	1	rs80356880
NM_007294.4(BRCA1): c.135-1G>T	Splicing defect	BC	4	1	rs80358158
NM_007294.4(BRCA1): c.181T>G	NP_009225.1: p.Cys61Gly	BC & OVC	5	2	no rs
NM_007294.4(BRCA1): c.191G>A	NP_009225.1: p.Cys64Tyr	BC	5	2	rs55851803
NM_007294.4(BRCA1): c.212G>A	NP_009225.1: p.Arg71Lys (Splicing defect)	BC	5	1	rs80356913
NM_007294.4(BRCA1): c.212+1G>A	Splicing defect	BC	5	1	rs80356913
NM_007294.4(BRCA1): c.415C>T	NP_009225.1: p.Gln139Ter	BC	6	2	rs80357372
NM_007294.4(BRCA1): c.431dup	NP_009225.1: p.Asn144LysfsTer15	BC	6	3	rs397509162
NM_007294.4(BRCA1): c.1016dup	NP_009225.1: p.Val340LysfsTer6	BC	10	1	rs80357569
NM_007294.4(BRCA1): c.1360_1361delAG	NP_009225.1: p.Ser454Ter	BC & OVC	10	5	rs80357969
NM_007294.4(BRCA1): c.1374del	NP_009225.1: p.Asp458GlufsTer17	BC	10	9	rs397508862
NM_007294.4(BRCA1): c.1504_1508delTTAAA	NP_009225.1: p.Leu502AlafsTer2	BC & colon ca	10	3	rs80357888
NM_007294.4(BRCA1): c.2008G>T	NP_009225.1: p.Glu670Ter	BC & OVC	10	2	no rs
NM_007294.4(BRCA1): c.2070_2073delAAGA	NP_009225.1: p.Lys690AsnfsTer10	BC	10	1	no rs
NM_007294.4(BRCA1): c.2568T>G	NP_009225.1: p.Tyr856Ter	BC	10	1	rs80356832
NM_007294.4(BRCA1): c.2597G>A	NP_009225.1: p.Arg866His	BC	10	1	rs80356911
NM_007294.4(BRCA1): c.2599G>T	NP_009225.1: p.Gln867Ter	BC	10	1	rs886038001
NM_007294.4(BRCA1): c.2641G>T	NP_009225.1: p.Glu881Ter	BC & OVC	10	28	rs397508988
NM_007294.4(BRCA1): c.3108del	NP_009225.1: p.Phe1036LeufsTer12	BC	10	1	rs80357841
NM_007294.4(BRCA1): c.3228_3229delAG	NP_009225.1: p.Gly1077AlafsTer8	BC	10	3	rs80357635
NM_007294.4(BRCA1): c.3288_3289delAA	NP_009225.1: p.Leu1098SerfsTer4	BC	10	1	rs80357686
NM_007294.4(BRCA1): c.3331_3334delCAAG	NP_009225.1: p.Gln1111AsnfsTer5	BC	10	1	rs80357701
NM_007294.4(BRCA1): c.3400G>T	NP_009225.1: p.Glu1134Ter	BC	10	1	no rs
NM_007294.4(BRCA1): c.3496_3497insT	NP_009225.1: p.Ala1166ValfsTer2	BC	10	1	no rs
NM_007294.4(BRCA1): c.3549_3550delAGinsT	NP_009225.1: p.Lys1183AsnfsTer27	Male BC	10	1	rs273899709
NM_007294.4(BRCA1): c.3593T>A	NP_009225.1: p.Leu1198Ter	Male BC	10	1	rs397509095
NM_007294.4(BRCA1): c.3732_3733delTA	NP_009225.1: p.His1244GlnfsTer10	BC	10	1	no rs
NM_007294.4(BRCA1): c.3756_3759delGTCT	NP_009225.1: p.Ser1253ArgfsTer10	BC & Melanoma	10	2	rs80357868
NM_007294.4(BRCA1): c.3947_3950delTCTT	NP_009225.1: p.Phe1316Ter	BC	10	2	rs886040177
NM_007294.4(BRCA1): c.4308_4309delTT	NP_009225.1: p.Ser1437CysfsTer3	BC	12	2	no rs
NM_007294.4(BRCA1): c.4327C>T	NP_009225.1: p.Arg1443Ter	BC	12	1	rs41293455
NM_007294.4(BRCA1): c.4524G>A	NP_009225.1: p.Trp1508Ter	BC	15	1	rs80356885
NM_007294.4(BRCA1): c.4838_4839insC	NP_009225.1: p.Pro1614SerfsTer8	BC	15	2	rs397509200
NM_007294.4(BRCA1): c.4868C>T	NP_009225.1: p.Ala1623Val	BC	15	1	no rs
NM_007294.4(BRCA1): c.4987-5T>A	Splicing defect	BC	16	1	rs397509214
NM_007294.4(BRCA1): c.5095C>T	NP_009225.1: p.Arg1699Trp	BC	17	2	rs55770810
NM_007294.4(BRCA1): c.5096G>A	NP_009225.1: p.Arg1699Gln	BC	17	3	rs41293459
NM_007294.4(BRCA1): c.5177_5180delGAAA	NP_009225.1: p.Arg1726LysfsTer3	BC	19	1	rs80357867
NM_007294.4(BRCA1): c.5229_5230delAA	NP_009225.1: p.Arg1744LysfsTer85	BC	19	4	rs80357852
NM_007294.4(BRCA1): c.5240_5243delGAAA	NP_009225.1: p.Arg1747LysfsTer3	BC	19	1	no rs
NM_007294.4(BRCA1): c.5266dup	NP_009225.1: p.Gln1756ProfsTer74	BC & OVC	19	6	rs80357906
NM_007294.4(BRCA1): c.5332+1G>C	Splicing defect	BC	21	1	rs80358041
NM_007294.4(BRCA1): c.5365_5366delGCinsA	NP_009225.1: p.Ala1789IlefsTer4	BC	21	1	no rs
NM_007294.4(BRCA1): c.5467+2T>G	Splicing defect	BC	21	3	rs80358009
NM_007294.4(BRCA1): c.5468-1G>A	Splicing defect	BC	23	1	rs80358048
**Total**				**143**	
***BRCA2***					
NC_000013.11:g.(?_32313776)_(32398795_?)del		BC	1 – 27	1	no rs
NC_000013.11:g.(?_32370334)_(32371115_?)del		BC & OVC	19 – 20	1	no rs
NM_000059.4(BRCA2): c.67+3A>G	Splicing defect	BC, Fanconi anemia	2	1	rs1593880835
NM_000059.4(BRCA2): c.93G>A	NP_000050.3: p.Trp31Ter	BC	3	1	rs80359214
NM_000059.4(BRCA2): c.516G>A	NP_000050.3: p.Lys172=	BC	6	2	rs80359790
NM_000059.4(BRCA2): c.582G>A	NP_000050.3: p.Trp194Ter	BC	7	13	rs80358810
NM_000059.4(BRCA2): c.771_775delTCAAA	NP_000050.3: p.Asn257LysfsTer17	BC	9	1	rs80359671
NM_000059.4(BRCA2): c.1261C>T	NP_000050.3: p.Gln421Ter	BC	10	1	rs80358419
NM_000059.4(BRCA2): c.1813dup	NP_000050.3: p.Ile605AsnfsTer11	Male BC	10	1	rs80359308
NM_000059.4(BRCA2): c.2636_2637delCT	NP_000050.3: p.Ser879Ter	BC	11	1	rs276174826
NM_000059.4(BRCA2): c.2806_2809delAAAC	NP_000050.3: p.Ala938ProfsTer21	BC	11	1	rs80359351
NM_000059.4(BRCA2): c.2826_2829delAATT	NP_000050.3: p.Ile943LysfsTer16	BC	11	1	rs397507643
NM_000059.4(BRCA2): c.2828_2831delTTAA	NP_000050.3: p.Ile943LysfsTer16	BC	11	1	rs397507643
NM_000059.4(BRCA2): c.3553dup	NP_000050.3: p.Thr1185AsnfsTer3	BC	11	1	no rs
NM_000059.4(BRCA2): c.3723del	NP_000050.3: p.Phe1241LeufsTer18	BC	11	1	rs886040491
NM_000059.4(BRCA2): c.3847del	NP_000050.3: p.Val1283LysfsTer2	BC	11	1	rs80359405
NM_000059.4(BRCA2): c.3881T>A	NP_000050.3: p.Leu1294Ter	BC	11	1	rs80358632
NM_000059.4(BRCA2): c.4003G>T	NP_000050.3: p.Glu1335Ter	BC	11	3	rs747070579
NM_000059.4(BRCA2): c.4456del	NP_000050.3: p.Val1486LeufsTer6	BC	11	1	no rs
NM_000059.4(BRCA2): c.4482_4483insAAAG	NP_000050.3: p.Ser1494LysfsTer20	BC & OVC	11	1	no rs
NM_000059.4(BRCA2): c.4568del	NP_000050.3: p.Gly1523ValfsTer20	BC	11	1	no rs
NM_000059.4(BRCA2): c.4936G>T	NP_000050.3: p.Glu1646Ter	BC	11	1	rs886038111
NM_000059.4(BRCA2): c.5082dup	NP_000050.3: p.Glu1695ArgfsTer5	BC	11	1	no rs
NM_000059.4(BRCA2): c.5213_5216delCTTA	NP_000050.3: p.Thr1738IlefsTer2	BC & OVC	11	2	rs80359493
NM_000059.4(BRCA2): c.5279C>G	NP_000050.3: p.Ser1760Ter	BC	11	1	rs80358751
NM_000059.4(BRCA2): c.5344C>T	NP_000050.3: p.Gln1782Ter	BC & OVC	11	1	rs80358757
NM_000059.4(BRCA2): c.5564C>G	NP_000050.3: p.Ser1855Ter	OVC	11	1	no rs
NM_000059.4(BRCA2): c.5771_5774delTTCA	NP_000050.3: p.Ile1924ArgfsTer38	Male BC, BC, OVC, prostate ca, endometrial ca, Fanconi aneamia	11	61	rs80359535
NM_000059.4(BRCA2): c.5946del	NP_000050.3: p.Ser1982ArgfsTer22	BC	11	5	rs80359550
NM_000059.4(BRCA2): c.6082_6086delGAAGA	NP_000050.3: p.Glu2028LysfsTer19	BC	11	1	rs80359558
NM_000059.4(BRCA2): c.6228del	NP_000050.3: p.Lys2077ArgfsTer4	BC	11	1	no rs
NM_000059.4(BRCA2): c.6393_6396delATTA	NP_000050.3: p.Lys2131AsnfsTer5	BC	11	1	rs397507849
NM_000059.4(BRCA2): c.6393del	NP_000050.3: p.Lys2131AsnfsTer6	BC	11	1	rs886038145
NM_000059.4(BRCA2): c.6447_6448dupTA	NP_000050.3: p.Lys2150IlefsTer19	BC & OVC	11	5	rs397507858
NM_000059.4(BRCA2): c.6623del	NP_000050.3: p.Asn2208IlefsTer2	BC	11	1	rs886038150
NM_000059.4(BRCA2): c.6937+2delT	Splicing defect	BC	12	1	no rs
NM_000059.4(BRCA2): c.7934del	NP_000050.3: p.Arg2645AsnfsTer3	Male BC, BC, OVC, prostate cancer, melanoma	17	176	rs80359688
NM_000059.4(BRCA2): c.7955T>G	NP_000050.3: p.Val2952Gly	BC	17	2	rs1555286868
NM_000059.4(BRCA2): c.8067T>A	NP_000050.3: p.Cys2689Ter	BC	18	4	rs80359046
NM_000059.4(BRCA2): c.8165C>G	NP_000050.3: p.Thr2722Arg	BC	18	1	rs80359062
NM_000059.4(BRCA2): c.8167G>C	NP_000050.3: p.Asp2723His	BC	18	3	rs41293511
NM_000059.4(BRCA2): c.8168A>T	NP_000050.3: p.Asp2723Val	BC	18	1	rs41293513
NM_000059.4(BRCA2): c.8331+2T>C	Splicing defect	BC	18	1	rs309122602
NM_000059.4(BRCA2): c.8504C>G	NP_000050.3: p.Ser2835Ter	BC	18	1	rs80359102
NM_000059.4(BRCA2): c.8686del	NP_000050.3: p.Arg2896ValfsTer13	BC	21	2	no rs
NM_000059.4(BRCA2): c.8696_8712del17	NP_000050.3: p.Gln2899LeufsTer2	BC & OVC	21	1	no rs
NM_000059.4(BRCA2): c.8754+1G>A	Splicing defect	BC	21	4	rs397508006
NM_000059.4(BRCA2): c.8954-2A>C	Splicing defect	BC	23	1	rs1135401928
NM_000059.4(BRCA2): c.8961_8964delGAGT	NP_000050.3: p.Ser2988PhefsTer12	BC	23	2	rs80359734
NM_000059.4(BRCA2): c.9105T>G	NP_000050.3: p.Tyr3035Ter	BC	23	3	rs886040819
NM_000059.4(BRCA2): c.9117G>A	NP_000050.3: p.Pro3039= (Splicing defect)	BC	23	1	rs28897756
NM_000059.4(BRCA2): c.9138del	NP_000050.3: p.Gln3047ArgfsTer15	BC	24	3	no rs
NM_000059.4(BRCA2): c.9154C>T	NP_000050.3: p.Arg3052Trp	BC	24	1	rs45580035
NM_000059.4(BRCA2): c.9351del	NP_000050.3: p.His3117GlnfsTer3	BC	25	8	no rs
NM_000059.4(BRCA2): c.9382C>T	NP_000050.3: p.Arg3128Ter	BC	25	1	no rs
NM_000059.4(BRCA2): c.9833_9842del	NP_000050.3: p.Pro3278HisfsTer32	BC	25	1	no rs
NM_000059.4(BRCA2): c.9435_9436delGT	NP_000050.3: p.Ser3147CysfsTer2	BC & OVC	25	5	rs80359763
**Total**				**342**	

Both the number of variants and their mutation spectrum differed for the various population groups. From the NGS data, it seemed as if the Black African group (*n* = 479) exhibited the largest diversity in both actionable and novel variants (44/479), as approximately double the number of variants were observed compared to the other groups (Asians 12/180; Caucasians 25/138; mixed ancestry 22/167 and individuals of unknown ethnicity 14/118). These numbers, however, do not accurately reflect the contribution of pathogenic variants to this group, as considerably more Black African patients were tested (at a ratio of approximately 3:1). Despite their higher diversity of pathogenic variants, the Black African group had the second-lowest positive detection rate (9.2%), apart from the Asian population with 6.6%. This can partly be attributed to patients being referred for genetic testing based on an early age at diagnosis alone, as most Black African patients were unaware of the accumulation of cancer occurrences in their families. In contrast, the Caucasian population exhibited the highest detection rate, namely 18.1%, despite a much lower number of patients tested using NGS. These patients seemed to be more appropriately selected as the majority of patients reported an intermediate to strong family history of BC and/or OVC. As the majority of mutation-positive patients carried one of the Afrikaner founder variants, the contribution of the Afrikaner founder variants to this group was evident.

The Caucasian and Black African detection rates declined from 18.1% to 9.2% to an overall 10% and 6.6%, respectively, once the founder variants detected during NGS were excluded. This finding indicates an ultimate positive mutation detection rate below 10% for NGS, which is currently not cost-effective. If these patients were screened using the first-tier targeted genotyping assay, costs could have been reduced by excluding these patients prior to NGS analysis. The difference in the positive detection rate between these two ethnic groups with well-characterized variants iterates the importance of family history and genetic cancer registries. By updating these registries, testing centers can keep track of related family members carrying actionable familial *BRCA1/2* variants, with the sole purpose to identify at-risk symptomatic-free family members.

This study attempted to report the mutation detection rates over the past two decades from a single institutional series’ perspective, with some biases due to: 1) the various techniques used; 2) disproportionate numbers of multiple ethnic groups studied; and 3) the minimum clinical criteria for BRCA testing changing. Therefore, the mutation detection rates presented per ethnic group does not accurately represent the positive predictive value of each technique and the national mutational burden of the respective groups. It merely reflects the frequencies of actionable variants detected at the time and within the performance specifications regarding the sensitivity and specificity of each mutation screening technique. The genotyping approach only identified selected pathogenic variants and did not enable the reporting of VUSes. Compared to NGS, screening for pathogenic variants using HRMA could have missed pathogenic variants and VUSes due to possible inadequate sensitivity during melt curve analysis in suboptimal PCR conditions (despite rigorous optimization and running each reaction in duplicate) (Combrink, 2016; Oosthuizen, 2016). In addition to a potential reduction in sensitivity of HRMA, the largest exons namely exon 10 of *BRCA1* and exons 10 and 11 of *BRCA2* were screened for protein-truncating variants only using PTT. The technique would therefore have missed various missense, synonymous or splice-site variants which could have represented actionable variants.

Sanger sequencing was utilized only for the confirmation of variants detected using the various mutation screening techniques. It was therefore not employed in this series for sequencing entire coding regions and splice site boundaries of samples. Moreover, biases in the detection frequencies of ethnic groups could have been introduced due to the disproportionate number of individuals in each group being screened with NGS, the most modern and sensitive technique in the test repertoire. Lastly, the criteria for *BRCA1/2* screening broadened with time and became more inclusive throughout the decades with more individuals currently meeting criteria than did a decade or two ago.

This series represents the most extensive report involving the *BRCA1/2* mutation spectrum on the African continent, surpassing the Nigerian study involving 1,136 patients (Supplementary Table S4). The positive mutation rate, however, was similar (9.2% versus a collective 11.1% in Nigeria), although the contribution of the genes was reversed, with *BRCA2* being more prevalent in SA (Supplementary Table S4). This observation can be attributed to the prevalence of three SA founder variants in *BRCA2*, representing three of the four SA ethnic groups (Black African, Caucasian and mixed ancestry groups). Although some African countries reported extremely high *BRCA1/2* mutation-positive rates above 15% (such as Egypt, Morocco, SA, Sudan and Tunisia), the majority of African studies involved small sample sizes based on very strict selection criteria.

Twenty-seven of the actionable variants were novel, with no unique identifier listed. The total number of novel variants increased when the complete variant list was considered (including benign variants to VUSes), with most present in a heterozygous form (Table 4). These novel variants were mostly observed for the Black African group, which was expected due to the high degree of variation evident in the African genome when compared to that of the Asian, African-American and European genomes. Recent genomic studies (Choudhury et al., 2020) revealed the presence of more than 3 million previously undescribed variants and predicted that only a fraction of the genetic diversity among individuals on the African continent has thus far been uncovered. This study exposed complex patterns of ancestral admixture, as both intra- and inter-population variations were observed. Although the authors did not observe a multitude of pathogenic variants in medically relevant genes, a significant number of variants denoted as likely-pathogenic in other genes were present in the ClinVar database (Choudhury et al., 2020). Such a high degree of genomic variation complicates the general approach of Mendelian classifications for variant interpretation, as for many variants, the MAF is either not known or very low, immediately classifying them as a rare variant, possibly also absent from population databases such as GnomAD and others.

This complexity highlights the necessity of functional assays performed in parallel with haplotype analysis. Haplotype inference for SA based on NGS data was performed by Oosthuizen et al. (2021). The authors reported several variants at low frequency to be in linkage disequilibrium in specific SA population groups, which emphasized the importance of long-range PCR confirmation for phasing. Suspected benign missense variants co-segregating with pathogenic variants or SNV-based VUSes, albeit at a low frequency, could act as potential modifiers regarding disease penetrance. Even subtle influences such as these can possibly contribute to the value of risk scores unique to population groups.

Collectively, these factors contribute to variants not being fully classified as actionable due to a lack of evidence (using the ACMG classification criteria). This resulted in potentially actionable variants currently classified as VUSes because of a paucity of evidence in the international literature. Finally, despite the increase in throughput and the extended scope for *BRCA1/2* variant discovery, less than 10% of patients with a personal history of cancer diagnosed at an early age, or who had a positive family history, received a positive test result, leaving the remainder of patients still in the dark regarding alternative management and therapeutic options involving poly (ADP-ribose) (PARP) inhibitors. As various other genes associated with *BRCA1/2* have been indicated to contribute to homologous recombination and DNA repair (such as ATM Serine/Threonine Kinase [*ATM*], BRCA1 Interacting Helicase I [*BRIP1*], Checkpoint Kinase 2 [*CHEK2*], RAD51 Paralog C [*RAD51C*] and Partner And Localizer of BRCA2 [*PALB2*]), the search for actionable variants responsible for hereditary BC and/or OVC needs to be expanded. With NGS already implemented, we propose moving towards multigene panel testing in the future. It could result in the identification of additional role players contributing to the disease burden in SA. By performing multipanel testing, we will be able to identify deleterious variants in multiple cancer susceptibility genes, which will allow us to identify eligible patients and related family members for clinical interventions, surveillance screening, targeted therapy and potential prevention strategies, according to the National Comprehensive Cancer Network (NCCN) guidelines.

## 5 Conclusion

The vision of better health systems for African countries is encompassed in the health-related sustainable development goals set by the World Health Organization (WHO) for Africa in 2018 (World Health Organization Africa, 2021). The SA Department of Health has recognized this initiative and pledged to reform this critical sector by releasing updated clinical guidelines for BC management and control during the same year. With the advances made in genetic testing for familial BC and OVC in state laboratories in SA, it has the potential to contribute immensely to the identification of high-risk *BRCA1/2* and non-*BRCA* germline actionable variants in patients. Given the magnitude of the disease, knowing a patient’s mutation status can aid in the individualization of their treatment, which is of great benefit for the attending physician as it contributes to the patient’s overall survival and will be of importance for related family members.

Due to the potential and far-reaching impact genetic testing has on augmenting the risk in a family with a positive family history of BC and/or OVC or in a patient diagnosed at an early age, it is imperative that the search is broadened to include other non-*BRCA* genes. Together, all these genes play an integral role in multiple signaling pathways inside the cell, with crosstalk between the associated proteins. If one of these signaling molecules becomes nonfunctional, the balance could be disturbed and may contribute to the progression of carcinogenesis.

The large number of novel and the abundance of heterozygous variants detected in this series reflect a high degree of genomic diversity. This highlights the existence of an immense gap in available naturally occurring population-specific knowledge due to a lack of African genomes in public genetic archives. Many diagnostic laboratories rely heavily on MAF and *in silico* predictions for variant interpretation and classification. In SA, as in many other African countries, this gap results in an unfavorable amount of VUSes classified. The lack of reference genomes increases the struggle to keep up with the rapid evolution in genetic variant screening for the confirmation of diagnosis. Although major strides have been made in the past decade in an attempt to catch up with first-world countries, uptake of genetic diagnostic services will not reach its full potential unless it becomes more affordable and a substantial number of African genomes is available to assist with variant interpretation and classification.

The SA scientific community is therefore compelled to continue with translational research in order to adopt first-world technologies into its healthcare context as a developing country. The vast progress made over the past two decades enabled a vital shift away from population-directed genetic testing to potentially comprehensive screening for all BC and OVC cancer patients. Consequently, the medical and scientific community in SA will continuously strive to comply with international standards and guidelines aimed at universal healthcare for all patients regardless of ethnicity, financial status or continent of birth. For centuries, the people of Africa have been marginalized and disadvantaged in many aspects, including optimal health care. With the WHO focusing on Africa, the health and well-being of its people are improving, resulting in the people of Africa currently sharing a vision for the future that is filled with optimism and hope.

## Data Availability

The datasets presented in this study can be found in online repositories. The names of the repository/repositories and accession number(s) can be found in the article/Supplementary Material.
