# Supplementary material for: Prevalence of Clinically Relevant Germline BRCA Variants in a Large Unselected South African Breast and Ovarian Cancer Cohort: A Public Sector Experience
Source: Front Genet. 2022 Apr 8;13:834265. doi: 10.3389/fgene.2022.834265 (PMC9024354; doi:10.3389/fgene.2022.834265)
Supplement: Supplementary file 1 [file Table1.docx]

**TABLE S1**: Correlation between the presence of a family history of BC and/or OVC and testing positive for an actionable *BRCA1/2* variant (reflected in percentages).

| **History classification** | **Mutation negative**  **(n=2 413)** | **Mutation positive**  **(n=481)** | **Total (%)** |
| --- | --- | --- | --- |
| No family history (n=916) | 34.3% | 18.5% | 31.7% |
| Unknown family history (n=141) | 5.8% | 0.0% | 4.9% |
| Family history (n=1 837) | 59.9% | 81.5% | 63.5% |
| **Total (%)** | **100.0%** | **100.0%** | **100.0%** |
