# Supplementary material for: Prevalence of Clinically Relevant Germline BRCA Variants in a Large Unselected South African Breast and Ovarian Cancer Cohort: A Public Sector Experience
Source: Front Genet. 2022 Apr 8;13:834265. doi: 10.3389/fgene.2022.834265 (PMC9024354; doi:10.3389/fgene.2022.834265)
Supplement: Supplementary file 2 [file Table2.docx]

**TABLE S2**: 455 BC patients divided according to the stage of disease at diagnosis (Stages 1 to 4) indicated per population group, reflected in percentages.

| **Ethnicity** | **Stage 1**  **(n=43)** | **Stage 2**  **(n=193)** | **Stage 3**  **(n=184)** | **Stage 4**  **(n=35)** | **Total (%)** |
| --- | --- | --- | --- | --- | --- |
| Asian (n=37) | 8.1% | 43.2% | 32.4% | 16.2% | 100.0% |
| Black African (n=132) | 6.1% | 41.7% | 44.7% | 7.6% | 100.0% |
| Caucasian (n=94) | 10.6% | 42.6% | 38.3% | 8.5% | 100.0% |
| Mixed ancestry (n=180) | 10.6% | 42.2% | 41.1% | 6.1% | 100.0% |
| Unknown (n=12) | 25.0% | 50.0% | 25.0% | 0.0% | 100.0% |
| **Total (%)** | **9.5%** | **42.4%** | **40.4%** | **7.7%** | **100.0%** |
