# Supplementary material for: Prevalence of Clinically Relevant Germline BRCA Variants in a Large Unselected South African Breast and Ovarian Cancer Cohort: A Public Sector Experience
Source: Front Genet. 2022 Apr 8;13:834265. doi: 10.3389/fgene.2022.834265 (PMC9024354; doi:10.3389/fgene.2022.834265)
Supplement: Supplementary file 3 [file Table3.docx]

**TABLE S3**: Comparison between patients affected with BC or OVC with regards to mutation status.

|  | **Unilateral BC**  **(n=1 867)** | **Bilateral BC**  **(n=220)** | **OVC**  **(n=91)** | **Other**  **(n=62)** |
| --- | --- | --- | --- | --- |
| No mutation | 1 574 | 183 | 71 | 61 |
| Mutation positive | 293 | 37 | 20 | 1 |
| Mutation positive rate (%) | 15.7 | 16.8 | 28.2 | 1.6 |

BC – breast cancer; OVC – ovarian cancer
