# Supplementary material for: Prevalence of Clinically Relevant Germline BRCA Variants in a Large Unselected South African Breast and Ovarian Cancer Cohort: A Public Sector Experience
Source: Front Genet. 2022 Apr 8;13:834265. doi: 10.3389/fgene.2022.834265 (PMC9024354; doi:10.3389/fgene.2022.834265)
Supplement: Supplementary file 4 [file Table4.docx]

**TABLE S4:** *BRCA1/2* mutation spectrum reported across the African continent.

| **Genes investigated** | **Country** | **Cancer type** | | **Sample size**  **(# of cases)** | **# of positives** | | **Positive mutation %** | **Reference** | |
| --- | --- | --- | --- | --- | --- | --- | --- | --- | --- |
| *BRCA1*  *BRCA2* | Algeria | BC & OVC | | 86 | *BRCA1* = 8  *BRCA2* = 2 | | 9.3%  2.3% | Cherbal *et al*., 2010 (51) | |
| *BRCA1*  *BRCA2* | Algeria | BC | | 40 | *BRCA1* = 4  *BRCA2* = 4 | | 10.0%  10.0% | Henouda *et al.,* 2016 (52) | |
| *BRCA1*  *BRCA2* | Algeria – West | BC & OVC | | 50 | *BRCA1* = 2  *BRCA2* = 2 | | 4.0%  4.0% | Boulenouar *et al.,* 2018 (53) | |
| *BRCA1*  *BRCA2* | Algeria – East | BC & OVC | | 113 | *BRCA1* = 3  *BRCA2* = 4 | | 2.7%  3.5% | Mehemmai *et al.,* 2019 (54) | |
| *BRCA1*  *BRCA2* | Burkina Faso | BC | | 51 | *BRCA1* = 5  *BRCA2* = 3 | | 9.8%  5.9% | Biancolella *et al.,* 2021 (55) | |
| *BRCA1*  *BRCA2* | Cameroon & Uganda | BC | | 196 | *BRCA1* = 11  *BRCA2* = 11 | | 5.6%  5.6% | Adedokun *et al.,* 2020 (56) | |
| *BRCA1*  *BRCA2* | Egypt | BC | | 50 | *BRCA1* = 9  *BRCA2* = 13 | | 18.0%  26.0% | Bensam *et al.*, 2014 (57) | |
| *BRCA1*  *BRCA2* | Egypt | OVC | | 104 | *BRCA1* = 15  *BRCA2* = 7 | | 14.4%  6.7% | Ashour & Ezzat Shafik, 2019 (58) | |
| *BRCA1* | Egypt | BC | | 80 | *BRCA1* = 4 | | 5.0% | Mogahed *et al.,* 2020 (59) | |
| *BRCA1*  *BRCA2* | Egypt | BC | | 103 | *BRCA1 =* 19  *BRCA2* = 17 | | 18.4%  16.5% | AbdelHamid *et al*., 2021 (60) | |
| *BRCA1*  *BRCA2* | Morocco | BC & OVC | | 40 | *BRCA1* = 5  *BRCA2* = 5 | | 12.5%  12.5% | Tazzite *et al.,* 2012 (61) | |
| *BRCA1* | Morocco | BC & OVC | | 121 | *BRCA1* = 7 | | 5.8% | Laraqui *et al.,* 2013 (62) | |
| *BRCA1* | Morocco | BC | | 71 | *BRCA1* = 2 | | 2.8% | El Khachibi *et al.,* 2015 (63) | |
| *BRCA1*  *BRCA2* | Morocco | BC & OVC | | 15 | *BRCA1* = 5  *BRCA2* = 1 | | 33.3%  6.7% | Jouali *et al.,* 2016 (64) | |
| *BRCA2* | Morocco | BC & OVC | | 122 | *BRCA2* = 14 | | 11.5% | Laarabi *et al.,* 2017 (65) | |
| *BRCA1*  *BRCA2* | Morocco – North | BC | | 33 | *BRCA1* = 3  *BRCA2* = 2 | | 9.0%  6.0% | Bakkach *et al.,* 2020 (66) | |
| *BRCA1*  *BRCA2* | Morocco | BC & OVC | | 64 | *BRCA1* = 12  *BRCA2* = 6 | | 18.8%  9.4% | El Ansari *et al.,* 2020 (67) | |
| *BRCA1*  *BRCA2* | Morocco | BC (TNBC) | | 32 | *BRCA1* = 6  *BRCA2* = 1 | | 18.8%  3.1% | Mansouri *et al.,* 2020 (68) | |
| *BRCA1*  *BRCA2* | Morocco | Prostate | | 30 | *BRCA1* = 1  *BRCA2* = 3 | | 3.3%  10.0% | Salmi *et al.,* 2021 (69) | |
| *BRCA1*  *BRCA2* | Nigeria | BC | | 70 | *BRCA1* = 3  *BRCA2* = 1 | | 4.3%  1.4% | Gao *et al.*, 2000 (70) | |
| *BRCA1* | Nigeria | BC | | 365 | *BRCA1* = 4 | | 1.1% | Zhang *et al.*, 2009 (71) | |
| *BRCA1*  *BRCA2* | Nigeria | BC | | 356 | *BRCA1* = 11  *BRCA2* = 0 | | 3.1%  0% | Zhang *et al.,* 2012 (72) | |
| *BRCA1*  *BRCA2* | Nigeria | BC | | 1 136 | *BRCA1* = 80  *BRCA2* = 47 | | 7.0%  4.1% | Zheng *et al.,* 2018 (73) | |
| *BRCA1* | Senegal | BC | | 27 | *BRCA1* = 15 | | 55.6% | Ndiaye *et al.,* 2020 (74) | |
| *BRCA1* | South Africa | BC & OVC | | 90 | *BRCA1* = 18 | | 20% | Reeves *et al.,* 2004 (6) | |
| *BRCA1* | South Africa | BC & OVC | | 51 | *BRCA1* = 15 | | 29.4% | Yawitch, 2005 (75) | |
| *BRCA1*  *BRCA2* | South Africa | BC & OVC | | 129 | *BRCA1* = 26  *BRCA2* = 43 | | 33.3%  20.2% | Schlebusch *et al.,* 2010 (76) | |
| *BRCA1*  *BRCA2* | South Africa | BC & OVC | | 121 | *BRCA1* = 2  *BRCA2* = 11 | | 1.7%  9.0% | van der Merwe *et al.*, 2012 (10) | |
| *BRCA1*  *BRCA2* | South Africa | BC (TNBC) | | 108 | *BRCA1* = 6  *BRCA2* = 7 | | 5.6%  6.5% | Francies *et al.,* 2015 (7) | |
| *BRCA1*  *BRCA2* | South Africa | BC | | 33 | *BRCA1* = 1  *BRCA2* = 2 | | 3.0%  6.0% | Chen, 2016 (22) | |
| *BRCA1*  *BRCA2* | South Africa | BC | | 54 | *BRCA1* = 4  *BRCA2* = 14 | | 7.4%  25.9% | Seymour *et al.,* 2016 (77) | |
| *BRCA1*  *BRCA2* | South Africa | BC & OVC | | 763 | *BRCA1* = 36  *BRCA2* = 49 | | 4.7%  6.4% | Oosthuizen *et al.,* 2021 (8) | |
| *BRCA1*  *BRCA2* | Sudan | BC | | 67 | *BRCA1* = 28  *BRCA2* = 2 | | 41.8%  3.0% | Melnour *et al.,* 2012 (78) | |
| *BRCA1*  *BRCA2* | Sudan | BC | | 35 | *BRCA1 =* 2  *BRCA2 =* 3 | | 5.7%  8.6% | Awadelkarim *et al.,* 2007 (79) | |
| *BRCA1* | Tunisia | BC & OVC | | 162 | *BRCA1* = 2 | | 1.2% | Mestiri *et al.,* 2000 (80) | |
| *BRCA1*  *BRCA2* | Tunisia | BC | | 36 | *BRCA1* = 5  *BRCA2* = 2 | | 13.9%  5.6% | Troudi *et al.,* 2007 (81) | |
| *BRCA1* | Tunisia | BC | | 9 | *BRCA1* = 2 | | 22.2% | Troudi *et al.,* 2009 (82) | |
| *BRCA1* | Tunisia | BC | | 16 | *BRCA1* = 6 | | 37.5% | Maufoudh *et al.,* 2012 (83) | |
| *BRCA1*  *BRCA2* | Tunisia | BC & OVC | | 66 | *BRCA1* = 8  *BRCA2* = 4 | | 12.0%  6.0% | Fourati *et al.,* 2014 (84) |  |
| *BRCA1*  *BRCA2* | Tunisia | BC & OVC | | 48 | *BRCA1* = 8  *BRCA2* = 4 | | 16.7%  8.3% | Riahi *et al.*, 2014 (85) |  |
| *BRCA2* | Tunisia | BC | 158 | | *BRCA2* = 1 | 0.6% | | Hadiji-Abbes *et al.,* 2015 (86) |  |
| *BRCA1*  *BRCA2* | Tunisia | BC | 17 | | *BRCA1* = 1  *BRCA2* = 0 | 6.0%  0% | | Awatef & Kassab, 2015 (87) |  |
| *BRCA1*  *BRCA2* | Tunisia | BC & OVC | 92 | | *BRCA1* = 17  *BRCA2* = 10 | 18.5%  10.9% | | Riahi *et al.*, 2016 (88) |  |
| *BRCA1*  *BRCA2* | Tunisia | BC & OVC | 354 | | *BRCA1* = 21  *BRCA2* = 15 | 5.9%  4.2% | | Hamdi *et al.,* 2021 (89) |  |
| *BRCA1* | Tunisia | BC | 122 | | *BRCA1* = 9 | 7.4% | | Mighri *et al.,* 2020 (90) |  |
| *BRCA1*  *BRCA2* | Tunisia – South | BC & OVC | 134 | | *BRCA1* = 10  *BRCA2* = 9 | 7.5%  6.7% | | Ayed-Guerfali *et al.,* 2021 (91) |  |

BC – breast cancer; OVC – ovarian cancer; TNBC – triple-negative breast cancer; # – number
